# The First District Dental Society of New York

**Published:** 1886-12

**Authors:** 


### THE FIRST DISTRICT DENTAL SOCIETY OF NEW YORK.

Reported Expressly for the Independent Practitioner.

This Society held its regular monthly meeting on the evening of October 5th, at the rooms of the S. S. White Dental Depot, cor. 32d St. and Broadway, the President, Dr. Wm. Carr, in the chair. After some routine business, the President called for reports of committees.

Dr. C. F. W. Bödecker, chairman of the Clinic Committee, reported a very successful clinic in the amount of work done and in attendance, the latter being from ninety to one hundred persons.

Dr. Pitt presented a boy with erosion, near the edge of the gum, of the four upper incisors and the two lower central incisors, the teeth seeming to be of good material. He was advised to fill the teeth temporarily with oxy-phosphate.

Dr. Nelson, of Boston, exhibited a new pair of pliers, and also some rubber and cloth discs, for polishing, made out of ordinary rubber packing, which will wear better than rubber alone.

Dr. Shumway, of Plymouth, filled a large incisor, the cavity occupying the mesial and cutting surfaces of the tooth. The tooth was devitalized, the pulp-canal cleaned and filled with oxy-phosphate of zinc and cotton.

Dr. Starr exhibited a vulcanizer with a lever, by which the head is fastened upon the vulcanizer with only one screw. He also exhibited Dr. F. H. Lee's improved blow-pipe.

Dr. William J. Younger, of San Francisco, implanted a right upper incisor for an employee of the S. S. White Dental Manufacturing Co. The tooth had been out of the mouth about one and a half years, and the one inserted was extracted at Cooper Institute in the morning. Dr. Younger cut into the alveolus, and fitted the root of the tooth perfectly. The patient said it felt very comfortable, and had been doing good service during the evening.

Dr. Reese showed his method of inserting gold and amalgam fillings by first lining the cavity with oxy-phosphate, and while this is yet soft, inserting the first layer of gold or amalgam.

Dr. A. C. Mellville exhibited some prepared steel which possessed remarkable properties. When ordinary steel is heated to redness and then gradually cooled, it softens, but this remained as hard as glass. The doctor had some porcelain crowns which had been drilled out by means of a diamond, and the holes enlarged by this piece of steel. He also exhibited some new screw posts, and illustrated their manufacture. He also exhibited a pair of forceps for widening crowns. He showed a number of other instruments, such as chisels, excavators, rubber polishing points, and wheels impregnated with emery or corundum, etc.

Dr. C. F. W. Bödecker exhibited a plaster cast representing a double lower jaw, sent to this country by Dr. Keller, of Cologne, Germany.* He also exhibited some aluminum bronze for base plates of artificial teeth, sent by Prof. C. Sauer, of Berlin, Germany.

Dr. Geo. Evans exhibited some bridge-work, introducing a novel feature. It is made of a combination of metal and rubber, with a groove or recess cut upon the buccal or labial portion of the teeth, which will prevent the bands from slipping toward the gum. The bridge can be readily removed by the patient and replaced.

---

* The history and a photograph of this jaw will appear in a future issue of this journal.

Dr. J. M. Crowell also exhibited a piece of bridge-work, made out of twenty carat gold, enameled with his new body gum.

The President announced that a committee would be appointed for the purpose of arranging an anniversary meeting of the Society.

Dr. Wm. H. Atkinson moved that an invitation be extended to Dr. Younger to give another clinic, showing his method of implanting teeth.

The President called upon Dr. Horatio C. Merriam, who read an elaborate paper on "Gutta-percha and its Uses in Operative Dentistry." The essayist first described the sources, varieties and manufacture of gutta-percha, illustrating his remarks by many specimens, showing the different forms and qualities in which the article is found in the market. By a series of experiments he arrived at the conclusion that gutta-percha will spoil when in contact with the air, daylight or heat for any length of time, and advises that it be kept under water in a dark, cool place. He spoke of gutta-percha as a temporary filling, for which it was highly recommended. In shallow or inaccessible cavities he recommended that the cavity be filled with warm gutta-percha, which should be removed and trimmed to the required shape. The plug should then be moistened with an essential oil and pressed back into the cavity. The essential oil softens the periphery of the gutta-percha, and after drying this resembles a coating of oil paint. It was claimed that this softened coating tends to prevent the destruction of the gutta-percha.

The President then called upon Dr. Edward C. Kirk, who read a paper on "The Etiology of Erosion." The essayist believed the trouble to be purely chemical, but found, as did others, that the disease was most common in people with nervous temperaments. In all cases examined, Dr. Kirk found an extremely acid condition of the fluids of the mouth, and this was very marked under the upper lip, near the cervical portions of the incisor teeth, immediately after rising in the morning. The mechanical theory of erosion he entirely discarded.

Dr. Dwinelle said that the mechanical friction of a tooth-brush would not account for all cases of erosion. He referred to a case spoken of by him in a former meeting of this Society, where the erosion upon the enamel was in every possible direction, so that the mechanical theory could not account for it.

Dr. W. H. Atkinson congratulated Dr. Younger upon the success of his operation at the clinic.

Dr. Younger announced that he had made a specialty of implanting teeth, and had given up other dental operations entirely. He would investigate the physiology of it with the microscope, at some future time. He said that the chances for success in implantation are far better than those of replantation or transplantation. In replanting as well as transplanting a tooth, the socket is usually in an inflamed or necrotic condition. In implantation both the new socket and the tooth are healthy. Speaking about the operation at the clinic to-day, he said that the tooth which he had to use was not as favorable to the case as he would have liked to have it, as the pericemental covering of the roots was wanting in several places. Dr. Younger claims that the vitality of the pericementum is something unusual in comparison with other tissues, as a tooth which has been out of the mouth for many years will again become firm in a new socket by the revitalization of the pericementum.

